# Screening and Determination of Synthetic PDE-5 Inhibitors in Adulterated Sexual Enhancement Supplements

**DOI:** 10.3390/molecules27196737

**Published:** 2022-10-09

**Authors:** Ammar Abdulrahman Jairoun, Sabaa Saleh Al-Hemyari, Moyad Shahwan, Sa’ed H. Zyoud, Baharudin Ibrahim, Samer H. Zyoud

**Affiliations:** 1 School of Pharmaceutical Sciences, Universiti Sains Malaysia (USM), Pulau Pinang 11500, Malaysia; 2 Health and Safety Department, Dubai Municipality, Dubai 67, United Arab Emirates; 3 Pharmacy Department, Emirates Health Services, Dubai 2299, United Arab Emirates; 4 Department of Clinical Sciences, College of Pharmacy and Health Sciences, Ajman University, Ajman 346, United Arab Emirates; 5 Center of Medical and Bio-Allied Health Sciences Research, Ajman University, Ajman 346, United Arab Emirates; 6 Poison Control and Drug Information Center (PCDIC), College of Medicine and Health Sciences, An-Najah National University, Nablus 44839, Palestine; 7 Clinical Research Centre, An-Najah National University Hospital, Nablus 44839, Palestine; 8 Faculty of Pharmacy, Universiti Malaya, Kuala Lumpur 50603, Malaysia; 9 Department of Mathematics and Sciences, Ajman University, Ajman 346, United Arab Emirates; 10 Nonlinear Dynamics Research Center (NDRC), Ajman University, Ajman 346, United Arab Emirates; 11 School of Physics, Universiti Sains Malaysia (USM), Penang 11800, Malaysia

**Keywords:** sildenafil, sexual enhancement supplements, undeclared pharmaceutical ingredients, adulteration

## Abstract

This paper reports an important investigation and quantification of adulteration of sexual enhancement supplements with prescription medicines available in United Arab Emirates (UAE): tadalafil, sildenafil and vardenafil. A total of 158 sexual enhancement supplements were collected and analyzed in the current study. The samples were screened using REVERSE-phase liquid chromatography tandem high-resolution mass spectrometry/mass spectrometry (RP-HPLC-MS/MS). Of all sexual enhancements, 12.7% (95% CI: 7.4–18) contained undeclared sildenafil, 3.8% (95% CI: 0.78–6.81) contained undeclared tadalafil and 1.9% (95% CI: 0.25–4.05) contained undeclared vardenafil. Of all sexual enhancement supplements, 13.9% (95% CI: 8.5–19.4) contained significant concentrations of sildenafil, tadalafil or vardenafil. While the study found relatively low levels of undeclared pharmaceutical ingredients in the sexual enhancement dietary supplements available on the UAE market, it is likely that patients with ED tend to consume multiple such supplements daily, thereby exposing themselves to highly elevated cumulative levels.

## 1. Introduction

The popularity of over the counter (OTC) herbal dietary supplements used to treat erectile dysfunction (ED), a condition that is estimated to affect up to 320 million men globally by 2025, has recently increased [1]. Many men use such supplements to address the effects of ED, which can occur due to several medical conditions, including hypertension, diabetes or the normal physiological changes that come with ageing [2]. Furthermore, men may self-medicate with dietary supplements because they prefer not to discuss such sensitive health issues with their doctors or seek alternatives to drug-mediated sexual performance enhancement [3]. The numerous herbal remedies available on the market are generally advertised as providing improved erectile function and performance; however, while these supplements are claimed to be effective and safe, they are also likely to be adulterated with various pharmaceutical ingredients and their analogues [3]. While herbal dietary supplements in general are considered to be broadly safe, there is far less certainty regarding the presence of active pharmaceutical ingredients (APIs) in supplements designed to enhance sexual performance [4].

This illegal practice is not confined to poorly regulated markets. In the United States, 572 adulterated supplements were reported between 2007 and 2014 by the strict national drug authority—the US Food and Drug Administration (FDA); 41.6% of these were marketed as sexual performance enhancers [4]. Meanwhile, the ad hoc international internet fraud team reported that one in three of so-called herbal supplements aimed at enhancing male sexual performance contained the approved active ingredients vardenafil or sildenafil [5]. As an example, the product “Rock Hard for Men”, sold as an herbal supplement in Australia and produced by an unknown manufacturer, contains two marketed phosphodiesterase-5 (PDE-5) inhibitors and second-generation sulfonylurea glyburide [6]. A study on so-called natural sexual enhancement supplements sold on informal markets in Singapore found that 77% contained undeclared pharmaceuticals, while slightly over 50% had above-therapeutic doses of pharmaceutical adulterants [7]. Another study on sexual enhancers revealed that 18% were adulterated with naproxen, chloramphenicol or lidocaine [8]. Many mislabeled or counterfeit male sexual enhancement products, the majority adulterated with PDE-5I, are seized every in many regions of the world every year [9].

Men suffering from ED unwittingly risk their health when using these so-called natural sexual performance enhancers due to the potential for contamination or adulteration with pharmaceutical ingredients such as PDE-5 inhibitors or other approved erectile dysfunction products. Furthermore, such products generally lack appropriate of safety information and are likely to be manufactured in establishments that do not comply with good manufacturing practices. In addition, these products pose a risk to users, who often have comorbidities, such as cardiovascular disease or diabetes, implying polypharmacy; using such supplements has been associated with severe side effects as well as drug interactions [10].

The UAE and other national drug regulatory authorities require dietary or herbal supplements to be manufactured in plants that demonstrate that their production follows strict good manufacturing practice guidelines and that any new products are tested in government or approved laboratories. Nevertheless, cases have been reported of dietary supplements being approved and put on the market despite being contaminated with microorganisms (Consumer Products Safety Section [11,12,13]). While the ministry responsible for the regulation of these products has warned citizens about the associated risks of consuming the sexual enhancement products, such as serious side effects, they remain widely available, especially through illegal online suppliers. The Ministry of Health and Prevention (MOHP) has a list of unregistered supplements that are particularly dangerous for men with high blood pressure or heart conditions [14]. Meanwhile, the Dubai Municipality has issued warnings about numerous supplements touting sexual enhancement or even slimming effects that contain banned pharmaceutical ingredients and have severe side effects [15,16]. Against this backdrop, there are concerns about the prevalence in the UAE of unregistered dietary supplements aimed at sexual enhancement containing undeclared sildenafil and other PDE-5 inhibitors. While this issue is rooted in complex origins, due to the implications for public health and safety, investigating these supplements is paramount.

This study aims to investigate this issue by quantifying the presence of undeclared prescription drugs and pharmaceutical chemicals, specifically sildenafil, tadalafil and vardenafil, in dietary herbal supplements aimed at sexual enhancement that are available in the UAE. The results will provide a better picture of the composition of the most widely available sexual enhancers in the UAE, thereby allowing current regulations to be strengthened and alternative methods to be developed to identify adulterants in sexual enhancement supplements, and thus, safeguard public health.

## 2. Results

### 2.1. Sample Description

A total of 158 sexual enhancement supplements were collected and analyzed in the current study. Of the total samples, 6 (3.8%) were manufactured in Canada, 24 (15.2%) in the European Union, 70 (44.3%) in the USA, 3 (1.9%) in Australia, 11 (7%) in China, 14 (8.9%) in India, 15 (9.5%) in Southeast Asia, 8 (5.1%) in the UAE and 7 (4.4%) had a nondeclared country of origin. Regarding the dosage forms, 65 (41.1%) were capsules, 18 (11.4%) were gelatin capsules, 13 (8.2%) were honey, 6 (3.8%) were liquid, 7 (4.4%) were powder and 49 (31%) were tablets (Table 1).

### 2.2. Assessment of Undeclared Active Pharmaceutical Ingredients in Herbal Sexual Enhancement Supplements

Of the 158 cases of sexual enhancement supplements, 15 (9.5%) included one undeclared prescription drug/chemical, and 7 (4.4%) included two undeclared prescription drugs/chemicals. Of all sexual enhancement supplements, 13.9% (95% CI: 8.5–19.4) contained significant concentrations of sildenafil, tadalafil or vardenafil (Table 2). Table 3 shows the prevalence of active pharmaceutical ingredients adulterants in sexual enhancement supplements in accordance with characteristics of each sample.

### 2.3. Comparison of Undeclared Prescription Drugs/Chemicals in Sexual Enhancement Supplements According to Sample Characteristics

Table 4 shows the presence and distribution of adulteration with active pharmaceutical ingredients and sample characteristics. The *p*-values estimates for the broad categories of dosage form and country of origin are also shown. The probability estimates were obtained using the chi-square and Fisher’s exact tests. The presence of undeclared active pharmaceutical ingredients was statistically significant associated with the labeled country of origin (*p* < 0.001). In addition, samples without a declared country of origin were more likely to contain undeclared active pharmaceutical ingredients majority adulterated with PDE-5 inhibitors. Similarly, a statistically significant association between marketed dosage forms and presence of active pharmaceutical ingredients adulterants (*p* = 0.03) was observed. Products formulated with honey vehicles were more likely to be adulterated with other active pharmaceutical ingredients.

## 3. Discussion

Any benefits offered by adulterated over the counter (OTC) sexually enhanced dietary supplements are far outstripped by the risks. As these adulterations frequently involve undeclared prescription drugs or other pharmaceutical chemicals, they can have adverse side effects in patients due to drug–drug interactions, particularly in patients with comorbidities, e.g., diabetes and heart disease, exposing them to polypharmacy. With this aspect in mind, this study examines the extent of availability on the UAE market of OTC sexual enhancement dietary supplements adulterated with prescription drugs and/or pharmaceutical chemicals. Of the 158 sexual enhancers tested, 13.9% contained undeclared pharmaceutical chemicals at various concentrations, including analogues of sildenafil, tadalafil or vardenafil.

This result is less than the result reported in previous studies. For example, in 80 samples analyzed in Iran, 23% were found to contain sildenafil [4]. Kerner [17] revealed that 80% of pills sold as “Viagra” online were in fact counterfeit and contained only 30–50% sildenafil, while a Dutch study on adulterated sexual enhancers seized by authorities found that almost 75% contained untested pharmaceuticals [8]. Similarly, in a French study examining sexual performance herbal supplements, PDE-5 inhibitors, including sildenafil, were identified in 61% of products [18], while a US study found that PDE-5 inhibited pharmaceuticals in 81% of 74 tested sexual enhancement products [10]. The findings in this study of lower levels of adulterants in sexual enhancers in the UAE may be due to its regulatory regime, whereby both the MOHP and the government require all available dietary supplements to be registered and adhere to strict safety, effectivity and quality regulations.

The most frequently detected adulterant detected in sexually enhanced dietary supplements sampled for the current study was sildenafil, followed by tadalafil and vardenafil. Overall, the levels of PDE-5 inhibitors were found to be 0.073 to 87,823 µg/day, which is consistent with prior research findings [4,17,19]. The consumption of adulterant pharmaceutical ingredients such as PDE-5 inhibitors is illegal in the UAE and puts consumers at risk of various adverse side effects, including headaches, dizziness, myalgia, dyspepsia, flushing and abnormal vision [20]. Beyond this factor, the significant heterogeneity found in the contents between the individual product samples implies that there is a lack of quality control in their production. Furthermore, most products were inadequately labeled, and different labels were often used for the same products [10].

Of particular concern is the finding that seven (4.4%) of the sexual enhancers sampled contained undeclared analogues of the PDE-5 inhibitors sildenafil and tadalafil. The diversity of these analogues found within individual samples represents a grave consumer risk and could cause fatalities, especially as these were not declared on the label and were difficult to identify. The difficulties that emerge in detecting and identifying these adulterants underline the need to increase the efforts to tackle adulteration in the name of public health [8,21]. Fortunately, numerous analytical methods exist with which to rapidly detect and structurally elucidate unidentified analogues and adulterants [8,21].

Moreover, PDE-5 inhibitors can have significant adverse effects on consumer health. For example, in the medically supervised prescription of medical quality sildenafil or tadalafil, the patients are made aware of the potential side effects, such as changes in vision or blood pressure decreases, and are instructed not to co-administer nitrates, as this could precipitate fatal blood pressure reductions. In contrast, the unintended consumption of PDE-5 inhibitors via dietary supplements, which have unknown dosage levels, can have unanticipated side effects as well as drug–drug interactions [22].

An additional concern in relation to sexual enhancement dietary supplements is the fact that they are widely advertised on television and online, especially on social media. A number of studies have found analogues of several PDE-5 inhibitors, including sildenafil, in adulterated herbal supplements that were advertised on television and online as enhancing sexual performance [23]. This issue underlines the need to strengthen public awareness of the risks posed by adulterated dietary supplements and promote the use of registered pharmaceuticals that do not have adverse, let alone fatal, health consequences [24,25].

Meanwhile, there is a clear need for the relevant authorities to take action by establishing a program for the pharmacovigilance of products marketed as sexual performance enhancers. Such an effort should incorporate the establishment of standard operating procedures (SOPs) as well as advertising, both online and on mainstream media. Furthermore, pharmacovigilance safety programs should be set up to gather a broad base on the toxicological characteristics of the sexual enhancement dietary supplements available on the market. Finally, exploring the reasons why consumers seek dietary supplements to improve their sexual performance would offer valuable insights that could drive the development of strategies to establish a pharmacovigilance system on a global scale [1].

Previous research has found that Chinese proprietary medicines produced in China as well as OTC drugs marketed as male sexual enhancement performers are frequently adulterated with sildenafil, tadalafil or vardenafil or their analogues [26]. Our results are in line with these findings, as PDE-5 inhibitors and their analogues emerged as frequent adulterants in Chinese-manufactured sexual enhancement supplements.

Notably, undeclared adulterants in the form of PDE-5 inhibitors were found more frequently in samples that used a honey-based formulation. This formulation is particularly concerning, as honey-based products are more likely to be consumed at levels above the recommended dose. Furthermore, the high demand for so-called herbal or natural dietary supplements, instead of prescribed medicines, is primarily due to the stigma that stems from ED, meaning patients are less likely to seek professional medical treatment. However, the consumption of OTC sexual enhancers adulterated with PDE-5 inhibitors that are marketed as a natural alternative to treating ED exposes patients to dangerous levels of these pharmaceutical drugs [10].

The increased availability of counterfeit and/or adulterated sexual enhancement supplements is becoming a global concern, underlining the need for patients to receive professional advice from the suppliers of these pharmaceuticals prior to purchase. Meanwhile, establishing a global database of known adulterated products and relevant analytical techniques via collaboration between governments and health regulatory authorities would enable illegal and unsafe products to be identified quickly and removed from the market. As long as the stigmatization of ED remains, patients are likely to continue to seek OTC supplements instead of medical advice to enhance their sexual performance, meaning that demand from the consumer is unlikely to abate on its own [25]. In addition, existing national pharmacovigilance monitoring systems should incorporate herbal or natural supplements, especially those claiming to enhance sexual performance, to draw public attention to the adverse effects of their use [26].

## 4. Materials and Methods

### 4.1. Chemicals

**Figure 1:**
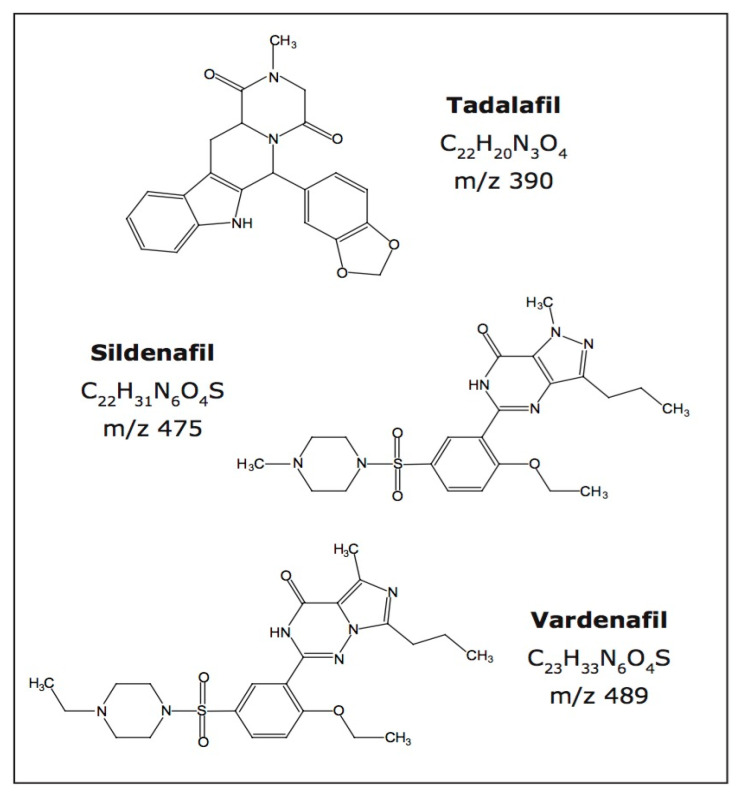
Chemical structure of sildenafil, tadalafil and vardenafil.

Standards of sildenafil citrate (CAS#171599-83-0), tadalafil (CAS#171596-29-5) and vardenafil (CAS#224785-90-4) (Figure 1) and the internal standards (ISTD) of sildenafil-d8 (CAS#951385-68-5), tadalafil-d3 (CAS#960226-55-5) and vardenafil-d5 (CAS#1189685-70-8) were from Dr. Ehrenstorfer, UK. HPLC grade (Merck, Kenilworth, NJ, USA) acetonitrile, formic acid, ammonium formate and methanol were used. Deionized water was used obtained from the Merck Milli-Q water purification system (Kenilworth, NJ, USA).

### 4.2. Sampling Method (Sample Collection)

Search efforts were focused on the local business directories to determine the facilities distributing dietary supplements aimed at sexual enhancement. The types of the researched premises included pharmacies and para-pharmacies, as well as food shops and healthcare stores. As a result, a total of 1500 facilities were located. The sampling framework was developed by using an Excel spreadsheet to organize all relevant information on these facilities, allowing their categorization and grouping by names, locations or addresses, contact phone numbers and e-mails. According to the inclusion criteria, a simple random-sample selection of the chosen facilities was conducted, using the facilities’ ID numbers stratified based upon the facilities’ location and type. At each of the chosen facilities, a single random packet of sexually enhanced dietary supplements was selected, regardless of the manufacturer or the country of the packet’s origin. For tracking purposes, each of the selected packets was assigned with a unique identifier, also helping to exclude sample supplication. Detailed information for every packet was recorded, focusing on the name of the brand, product title, manufacturer, country of origin, product category, subcategory, batch number, bar code, volume or size (when appropriate), form of the product, the dosage of a single packet, the recommended dosage for the whole pack and the address or location of the facility where the packet was bought. Any two items under the same name but in different forms (e.g., tablets, capsules and honey) or manufactured by different organizations were regarded as different and tested accordingly. All packets underwent a lab analysis on the day of their collection.

### 4.3. Sample Treatment

The homogenization of capsules and tablets into fine powders was performed with the use of a mortar, grinder and pestle. The sample was then prepared from the homogenized material. The homogenization of liquids and powders was performed by stirring them with a glass rod or a spatula. The sample was later prepared with the use of homogenized material. A blender was exploited for the homogenization of the samples that contained the herbal tea-like leaves and roots. The sample was later prepared from the homogenized material.

About 0.5 g of every homogenous sample derived from each food, dietary supplement, and health supplement were added into a 20 mL stoppered flask. Later, to 400 µL of ISTD intermediate solution mix (1 ppm), 10 mL of acetonitrile/water (50:50, *v*/*v*) was added and then sonicated to complete disintegration. The mixture was then cooled to room temperature and, with the use of diluent, diluted to the relevant volume. The separation of the two phases was completed by centrifugation (6000 rpm, 20 min), after which the filtration of the supernatant solution was completed through a 0.2 µm nylon syringe filter to put it into an HPLC autosampler vial.

### 4.4. RP-HPLC-MS/MS Analysis

The RP-HPLC-MS/MS) analysis was conducted using Agilent 1260 infinity series 6420 Triple Quad MS (Agilent, 3000 Hanover, Palo Alto, CA, USA) system. The column used was Kinetex XB-C18 (100 Å, 50 × 2.1 mm). Table 5 presents the gradient concentrations of mobile phases consisting of varying concentrations of A (3 mM ammonium formate + 0.1% formic acid in water) and B (0.1% formic acid in acetonitrile) at the gradients shown. The injection volume was 5 μL at 0.30 mL/min and column temperature maintained at 50 °C to achieve chromatographic separation. The identification of the peaks of the sample components was based on their mass and contrasting their retention time to the standard retention time. The run time was estimated at 15 min, as shown in the tables below (Table 5 and Table 6).

### 4.5. Validation Methodology

Successful method validation was done in accordance International Conference on Harmonization (ICH) guidelines. The method’s accuracy, precision, linearity and the limits of detection and quantification (LOD and LOQ) were determined. The chromatographic peak resolution received using sildenafil, tadalafil and vardenafil fostered method selectivity.

The calibration standards for sildenafil, tadalafil and vardenafil at 1.0, 5.0, 10.0, 50.0, 100 and 200.0 µg/kg and the ISTD of sildenafil-d8, tadalafil-d3 and vardenafil-d5 at the 20.0 µg/kg concentration were spiked into the study blank matrix, i.e., the analyte-free matrix. The extraction of the added blank samples occurred in the 20 mL stoppered volumetric flask for further analysis that was to be completed after the above-described sample preparation procedures. Long-term storage of the solutions was at −20 °C in amber-colored glass vials. The method’s linearity was assessed between concentrations of 1.0–200.0 µg/kg with an R^2^ value >0.995.

The determination of LOD was performed using the blank samples (including dietary supplements, e.g., tablets, capsules and honey) with at least a 3:1 signal-to-noise ratio.

The estimation of LOQ was completed according to a S/N ratio of a minimum 10. The upper and lower limit of 5.0–100.0 µg/kg was used to test the method’s LOD and LOQ. Test results demonstrated the limits at 10.0 µg/kg for LOD and 40.0 µg/kg for LOQ.

The injection of the standard mixtures of HE 3 analytes with internal standard at concentrations of 1.0 µg/kg, 10 µg/kg and 150 µg/kg was completed six times per day for the repeatability test to estimate the peak area and retention time (RT). The relative standard deviation (RSD) of the intraday precision was determined at 0.6% for RT and 8.2% for peak area.

Accuracy and precision were successfully conducted by spiking six separate solutions at LOQ, medium concentration and high concentration levels compared to the strengths used for calibration. To validate this method, sildenafil, tadalafil and the indicator were spiked at 1.0 µg/kg, 10 µg/kg and 150 µg/kg in the blank products, including dietary supplement tablets, capsules and honey, with further preparation and analysis for all the six spike concentration levels. The findings on % RSD and % Recoveries were presented in Table 7.

Furthermore, the injections of the calibration mixtures of three analytes with internal standard (20 µg/kg) at 1.0 µg/kg (low), 10 µg/kg (medium) and 150 µg/kg (high) concentrations, six times on consecutive days was performed for the reproducibility test in the analyte-free products, including dietary supplement tablets, capsules and honey, with their further preparation and analysis of all six concentration levels. The findings on % RSD and % Recoveries are shown in Table 8.

To assure the quality of the analytical procedure (QA/QC), 10 mg of sildenafil, tadalafil and vardenafil were added to methanol solvent into a 10 mL stoppered flask to obtain a 1000 mg/L concentrated solution. After that, serial dilutions of the stock solutions to 20 µg/L were done with methanol as the solvent. The final stock solution was stored in amber-colored glass vials at −20 °C. The standard-based quality control test was performed by using the 10 µg/L solution of the separately diluted standard developed from the same or different lot, with 90–110% recovery. For quality control of samples, the blank matrix was spiked to achieve a concentration of 10 µg/kg, and 80–120% recovery. Test samples were analyzed in duplicate to research duplicate sample preparation. The samples for testing were spiked to a concentration of 10 µg/kg, with 80–120% recovery, to assess the preparation of the spiked samples. The injection of the study standard 10 µg/kg preparation, with 90–110% recovery, was completed at the end of the injection sequence to check standards.

Sample quantifications for the three PDE-5 inhibitors were calculated using the intensity of the ratio of their two major fragment ions. The calculation of the peak area ratio (PAR) for each working solution preparation was performed by dividing the drug peak area: sildenafil, tadalafil and vardenafil (AREA sildenafil, tadalafil and vardenafil) by the internal standard’s peak area (AREAIS). The construction was achieved based on the calibration curves obtained from the PAR and the known concentration of the prepared standard solutions. The following formula was used to calculate sildenafil, tadalafil and vardenafil concentrations in the unknown samples:

Concentration (mg/kg) = Instrument Con. (µg/kg) × Make up volume (mL) × Dilution ÷ Weight of sample (g) × 1000(1)

### 4.6. Ethical Considerations

The Institutional Review Board of An-Najah National University Approved the study (ref. Phd/2/20/16).

### 4.7. Statistical Analysis

The data analysis was performed using SPSS v.26 Chicago, IL. Frequencies and percentages were calculated to summarize the categorical variables. The incidence of hidden pharmaceutical ingredients in sexual enhancement supplements was categorized as the percentages of supplements that have quantifiable amounts of sildenafil, tadalafil or vardenafil. With every supplement product having unlabeled pharmaceutical ingredients, the active pharmaceutical ingredients concentration present as adulterants in the samples (mg/kg) and the approved dose per day (g) were used to calculate the recommended daily dosage intake of each pharmaceutical ingredient. To explore the relationship between incidence of hidden pharmaceutical ingredients and sample characteristics (dosage form and country of origin), we used Chi-square and Fisher’s precise tests and a sureness level of 95%.

## 5. Conclusions

While the study found relatively low levels of undeclared pharmaceutical ingredients in the sexual enhancement dietary supplements available on the UAE market, it is likely that patients with ED tend to consume multiple such supplements daily, thereby exposing themselves to very high cumulative levels. Hence, there is an urgent need to enact stricter laws and regulations that actively tackle the issue by stipulating how these products are registered, marketed and sold. Furthermore, the manufacturers of these products must be made to follow purity and marketing regulations. Finally, there should be continuing efforts to research, regulate, report on and educate on sexual enhancement supplements, leading to enhanced public health and safety.

## Figures and Tables

**Table 1:** Number and percentages of sample baseline characteristics (*n* = 158).

Characteristics	Groups	Frequency	Percentage
Dosage forms	Capsules	65	41.1%
	Gelatin capsules	18	11.4%
	Honey	13	8.2%
	Liquid	6	3.8%
	Powder	7	4.4%
	Tablets	49	31%
Country of origin	Canada	6	3.8%
	EU	24	15.2%
	USA	70	44.3%
	Australia	3	1.9%
	China	11	7%
	India	14	8.9%
	South East Asia	15	9.5%
	UAE	8	5.1%
	Undeclared	7	4.4%

**Table 2:** Estimation of hidden prescription drugs and chemicals in sexual enhancement supplements.

Prescription Drugs/Chemicals	Proportion of Sexual Enhancement Supplements That Contains Hidden Prescription Drugs/Chemicals			
	N	%	95% CI	
			Lower	Upper
Sildenafil	20	12.7%	7.4	18
Tadalafil	6	3.8%	0.78	6.81
Vardenafil	3	1.9%	0.25	4.05
Sildenafil, tadalafil or vardenafil	22	13.9%	8.5	19.4
Number of products that included undeclared prescription drugs/chemicals				
One	15	9.5%	-------	-------
Two	7	4.4%	-------	-------

Abbreviations: 95% CI; confidence interval; N, Number of Samples.

**Table 3:** List of tested sexual enhancement supplement products according to the prescription drugs/chemicals and sample characteristics.

Sample Characteristics			Concentration (mg/kg)			Daily Dose (µg/day)			Number of Hidden Chemicals
Sample Code	Country of Origin	Dosage Form	Sildenafil	Tadalafil	Vardenafil	Sildenafil	Tadalafil	Vardenafil	
1	EU	Tablets	0.58	<0.04	<0.04	2.76	---	---	1
2	USA	Capsules	0.069	<0.04	<0.04	0.073	---	---	1
3	China	Capsules	263,287	<0.04	<0.04	41,230.7	---	---	1
4	South East Asia	Honey	<0.04	775	<0.04	---	11,625	---	1
5	South East Asia	Honey	<0.04	824	<0.04	---	16,480	---	1
6	South East Asia	Honey	1300	<0.04	<0.04	26,000	---	---	1
7	UAE	Honey	2053	<0.04	<0.04	20,530	---	---	1
8	China	Tablets	647.9	<0.04	<0.04	428.77	---	---	1
9	China	Tablets	77,534	<0.04	<0.04	59,963.2	---	---	1
10	South East Asia	Powder	12.58	0.262	<0.04	25.16	0.524	---	2
11	China	Capsules	230,438	36,006	<0.04	55,618.52	8690.408	---	2
12	ND	Capsules	309,422	<0.04	<0.04	53,171.1	---	---	1
13	ND	Capsules	134	<0.04	<0.04	41.9	---	---	1
14	South East Asia	Capsules	54,250.9	<0.04	<0.04	41,823.2	---	---	1
15	UAE	Honey	4530	<0.04	<0.04	60,565	---	---	1
16	China	Tablets	7229.99	<0.04	<0.04	530.77	---	---	1
17	China	Tablets	97,620	<0.04	<0.04	79,563.3	---	---	1
18	South East Asia	Powder	12.58	0.262	<0.04	65.83	0.524	---	2
19	China	Capsules	542,200	36,006	<0.04	70,668	8690.41	---	2
20	ND	Capsules	568,700	<0.04	348,800	53,171.1	---	531.1	2
21	ND	Capsules	8120	<0.04	125,000	87.97	---	67.9	2
22	South East Asia	Capsules	78,200	<0.04	872,999.9	87,823.2	---	83.2	2

Notes: Daily dose calculated using test concentration of each chemical (mg/kg) and the manufacturer’s advised daily adult dose (g), NA; not declared.

**Table 4:** Comparison of hidden prescription chemicals and drugs according to the sample characteristics.

Characteristics	Groups	Hidden Prescription Chemicals and Drugs		
		N	%	*p*-Value
Dosage Form	Capsules	10	15.4	0.030 *
	Gelatin capsules	0	0	
	Honey	5	38.5	
	Liquid	0	0	
	Powder	2	28.6	
	Tablets	5	10.2	
Country of origin	Canada	0	0	<0.001 *
	EU	1	4.2	
	USA	1	1.4	
	Australia	0	0	
	China	7	63.6	
	India	0	0	
	South East Asia	7	46.7	
	UAE	2	25	
	Undeclared	4	57.1	

* p-value less than 0.005, *p*-value reported above for comparisons between variable level “category-levels” using the Fisher exact tests and Chi-square test, N, Number of products.

**Table 5:** Gradient program of the mobile phase and MS conditions.

Gradient Program			MS Conditions-Source Parameters
**Time**	**% Mobile Phase A**	**% Mobile Phase B**	Ion Source: ESI Scan Type: MRM Div. valve: 0.0 min-To waste 4.4 min-To MS 7.8 min-To waste Delta EMV(+): 400 Gas Temp: 350 °C N2 Gas flow: 10 L/min Nebulizer: 60 psi Capillary Voltage: 4000 V (Positive and Negative) Chromatogram: TIC
0.0	85.0	15.0	
0.5	85.0	15.0	
7.5	20.0	80.0	
8.0	20.0	80.0	
8.5	85.0	15.0	
13.0	85.0	15.0	

**Table 6:** Acquisition parameters.

Compound Name.	Precursor Ion	MS1 Res	Product Ion	MS2 Res	Dwell	FV	CE	CAV	Polarity
Vardenafil	489.6	Wide	312	Unit	25	182	40	7	Positive
		Wide	151 *	Unit	25	182	48	7	Positive
Sildenafil	475.6	Wide	100.1	Unit	25	164	24	7	Positive
		Wide	58.2 *	Unit	25	164	48	7	Positive
Tadalafil	390.4	Wide	268 *	Unit	25	134	8	7	Positive
		Wide	169.1	Unit	25	134	36	7	Positive
Vardenafil-d5	494.6	Wide	299.1	Unit	25	197	40	7	Positive
Sildenafil-d8	483.6	Wide	108.2	Unit	25	187	28	7	Positive
Tadalafil-d3	393.4	Wide	271.1	Unit	25	101	8	7	Positive

* Product ions were used for quantitation, Abbreviation: MS; Mass spectrometry, Dwell; Dwell time, FV; Fragmentor voltage, CE; Collisional energy, CAV; Cell accelerator voltage.

**Table 7:** Accuracy and Precision on day 1.

Matrix Name	% RSD of RT	Spike at 1.0 µg/kg		Spike at 10 µg/kg		Spike at 150 µg/kg	
		% RSD	% Recovery	% RSD	% Recovery	% RSD	% Recovery
Dietary supplement-tablet	1.2	16.2	82.9	9.7	88.1	7.2	90.8
Honey	0.8	10.6	89.3	7.5	90.5	5.0	93.4
Dietary supplement-capsule	1.9	14.8	83.8	8.3	87.7	6.6	92.5

Abbreviation: RSD; relative standard deviation, RT; Retention time.

**Table 8:** Accuracy and Precision on day 2 (Reproducibility).

Matrix Name	% RSD of RT	Spike at 1.0 µg/kg		Spike at 10 µg/kg		Spike at 150 µg/kg	
		% RSD	% Recovery	% RSD	% Recovery	% RSD	% Recovery
Dietary supplement-tablet	1.4	15.3	84.0	10.5	90.3	8.4	89.1
Honey	1.0	11.8	87.4	8.8	91.7	7.0	90.8
Dietary supplement-capsule	1.7	16.2	85.9	11.1	86.0	7.8	94.7

Abbreviation: RSD; relative standard deviation, RT; Retention time.

## Data Availability

Not applicable.
